# Dexmedetomidine and acute kidney injury following cardiac surgery in pediatric patients—An updated systematic review and meta-analysis

**DOI:** 10.3389/fcvm.2022.938790

**Published:** 2022-08-24

**Authors:** Hongbai Wang, Chaobin Zhang, Yinan Li, Yuan Jia, Su Yuan, Jianhui Wang, Fuxia Yan

**Affiliations:** ^1^Department of Anesthesiology, Fuwai Hospital, Chinese Academy of Medical Sciences and Peking Union Medical College, Beijing, China; ^2^Department of Anesthesiology, Fuwai Hospital, Chinese Academy of Medical Sciences, Shenzhen (Sun Yat-sen Cardiovascular Hospital, Shenzhen), Shenzhen, China

**Keywords:** pediatric patients, congenital heart disease, dexmedetomidine, cardiac surgery, cardiopulmonary bypass, acute kidney injury, meta-analysis

## Abstract

**Background:**

Acute kidney injury (AKI) is a common postoperative complication in pediatric patients undergoing cardiac surgery and associated with poor outcomes. Dexmedetomidine has the pharmacological features of organ protection in cardiac surgery patients. The aim of this meta-analysis is to investigate the effect of dexmedetomidine infusion on the incidence of AKI after cardiac surgery in pediatric patients.

**Methods:**

The databases of Pubmed, Embase, and Cochrane Library were searched until April 24, 2022 following the Preferred Reporting Items for Systematic Reviews and Meta-Analyses (PRISMA) guidelines. RevMan 5.3 was used to perform statistical analyses.

**Results:**

Five relevant trials with a total of 630 patients were included. The pooled result using fixed-effects model with OR demonstrated significant difference in the incidence of AKI between patients with dexmedetomidine and placebo (OR = 0.49, 95% CI: [0.33, 0.73], *I*^2^ = 0%, *p* for effect = 0.0004). Subgroup analyses were performed based on congenital heart disease (CHD) types and dexmedetomidine intervention time. Pooled results did not demonstrate considerable difference in the incidence of AKI in pediatric patients receiving intraoperative (OR = 0.53, 95% CI: [0.29, 0.99], *I*^2^ = 0%, *p* for effect = 0.05) or postoperative dexmedetomidine infusion (OR = 0.56, 95% CI: [0.31, 1.04], *p* for effect = 0.07), but a significant difference in patients receiving combination of intra- and postoperative dexmedetomidine infusion (OR = 0.27, 95% CI: [0.09, 0.77], *p* for effect = 0.01). Besides, there was no significant difference in duration of mechanical ventilation (SMD: –0.19, 95% CI: –0.46 to 0.08, *p* for effect = 0.16; SMD: –0.16, 95% CI: –0.37 to 0.06, *p* for effect = 0.15), length of ICU (SMD: 0.02, 95% CI: –0.41 to 0.44, *p* for effect = 0.93) and hospital stay (SMD: 0.2, 95% CI: –0.13 to 0.54, *p* for effect = 0.23), and in-hospital mortality (OR = 1.26, 95% CI: 0.33–4.84, *p* for effect = 0.73) after surgery according to the pooled results of the secondary outcomes.

**Conclusion:**

Compared to placebo, dexmedetomidine could significantly reduce the postoperative incidence of AKI in pediatric patients undergoing cardiac surgery with cardiopulmonary bypass (CPB), but the considerable difference was reflected in the pediatric patients receiving combination of intra- and postoperative dexmedetomidine infusion. Besides, there was no significant difference in duration of mechanical ventilation, length of ICU and hospital stay, or in-hospital mortality after surgery.

## Introduction

Acute kidney injury (AKI) is a common postoperative complication in pediatric patients undergoing cardiac surgery with cardiopulmonary bypass (CPB) due to congenital heart disease (CHD). The incidence of AKI ranges from 20 to 86% depending on different diagnostic tools and medical centers (1). The occurrence of AKI is a risk factor for adverse prognosis, such as prolonged ICU and hospital stay, and increased mortality (2, 3). Studies demonstrated that the elevated reactive oxygen species, hemolysis and systemic inflammatory response induced by CPB have been suggested to be the potential mechanisms developing pediatric AKI after cardiac surgery (4, 5). Additionally, several risk factors of AKI have been identified, such as younger age, complicated cyanotic CHD, perioperative hemodynamic compromise, longer CPB time, fluid overload (6, 7).

Dexmedetomidine is a highly selective α_2_ -adrenergic receptor agonist, and it also has the pharmacological features of sedation, analgesia, systemic circulation stability, and anti-inflammatory response (8). Therefore, dexmedetomidine has the potential effect of nephroprotection in pediatric patients undergoing cardiac surgery. Although dexmedetomidine is still considered as off-label use in pediatric patients, piles of clinical studies on effect of dexmedetomidine on organ protection in children have been completed or are on progress (9, 10). The primary aim of this systematic review and meta-analysis was to investigate the effect of dexmedetomidine infusion on the incidence of AKI after cardiac surgery in pediatric patients by synthesizing the results of previous clinical studies.

## Methods

We performed this systematic review and meta-analysis according to the guidelines of the 2009 Preferred Reporting Items for Systematic Reviews and Meta-Analyses (PRISMA) (Supplementary Table 1) (11).

## Search strategy

Two authors were independently responsible for document retrieval. We searched the databases of Pubmed, Embase, and Cochrane Library using the PICOS (Population, Intervention, Comparison, Outcome, Study design) method. Our last search was completed on April 24, 2022. The search terms included “pediatric” OR “pediatrics” OR “child” OR “children” OR “infant” OR “infants” OR neonate” OR “neonates” OR “newborn” OR “newborns” OR “teenager” OR “teenagers” AND “cardiac surgery” OR “cardiac operation” OR “cardiac surgeries” OR “heart surgery” OR “heart surgeries” OR “heart operation” AND “acute kidney injury” OR “acute renal injury” OR “acute kidney failure” OR “acute renal failure” OR “acute kidney insufficiency” OR “acute renal insufficiency” AND “dexmedetomidine” OR “MPV-1440” OR “MPV 1440” OR “MPV1440” OR “Precedex” OR “dexmedetomidine Hydrochloride,” and the search scope was “title and abstract.” Because we sought to examine all studies about the effect of dexmedetomidine on the incidence of AKI in pediatric patients undergoing cardiac surgery, we did not constrain the search terms for study designs.

## Study selection

Two authors conducted the screening process for titles and abstracts, while another two authors performed the screening process for full texts. The inclusion criteria were (1) participants aged younger than 18 years; (2) patients undergoing cardiac surgery; and (3) articles reporting the effect of dexmedetomidine on AKI. The exclusion criteria were: (1) duplicate articles; (2) participants older than 18 years old; (3) review or meta-analysis; (4) articles published as an abstract, letter, case report, basic research, editorial, note, method, or protocol; (5) articles presented in a non-English language; (6) studies without a specific number of patients with dexmedetomidine (observational studies) and/or AKI.

## Quality assessment of included studies

Two authors independently assessed the quality of included studies. For RCTs, we assessed the risk of bias using the Cochrane Collaboration Risk of Bias Assessment tool, which included the following seven items: (1) random sequence generation, (2) allocation concealment, (3) blinding of participants and personnel, (4) blinding of outcome assessment, (5) incomplete outcome data, (6) selective reporting, and (7) others (bias due to vested financial interest and academic bias) (12). If a trial was found to have one or more of the items associated with high or unclear risk of bias, this trial was classified as high risk. For the observational trials, risk of bias was assessed using the Newcastle–Ottawa Quality Assessment Scale (NOS), which comprises the following three domains: (1) selection, (2) comparability, and (3) outcome for cohort studies (13). There were four stars in the selection domain, two stars in the comparability domain, and three stars in the exposure domain. Trials with seven or more cumulative stars were considered to be of high quality, those with six stars of moderate quality, and those with < 6 stars of low quality. If the two authors disagreed on their assessment, they consulted the third or fourth author. Eventually, we reached a consensus.

## Data extraction

Two authors were responsible for extracting the following information: (1) authors; (2) publication year; (3) country of publication; (4) total number of participants in each study; (5) percentage of males; (6) age range of all the participants; (7) weight; (8) procedures that the participants underwent; (9) dose and time points of dexmedetomidine administration; (10) duration of CPB; (11) duration of aortic clamping; (12) assessment time of AKI; (13) number of patients with or without dexmedetomidine; (14) assessment (or follow-up) time; (15) number of patients with and without AKI; (16) postoperative duration of mechanical ventilation; (17) postoperative length of ICU stay; (18) postoperative length of hospital stay; (19) number of dead patients during the follow-up time. Another two authors were responsible for adjusting data discrepancies.

## Outcome measures

The primary aim of this meta-analysis was to investigate the effect of dexmedetomidine infusion on the incidence of AKI in pediatric patients undergoing cardiac surgery with CPB. Additionally, we also evaluated the association between dexmedetomidine infusion and duration of mechanical ventilation, length of ICU and hospital stay, and all-cause mortality after surgery as the secondary outcomes.

## Data analysis

Review Manager (RevMan) version 5.3 (Cochrane collaboration, Oxford, United Kingdom) was used to perform statistical analyses. We assessed the heterogeneity of included studies using the values of *I*^2^ and the Mantel-Haenszel chi-square test (*p*-value for heterogeneity). The values of *I*^2^ < 40%, *I*^2^ = 40–60%, and *I*^2^ > 60% indicated low, moderate, and high heterogeneity, respectively (14). If we identified *I*^2^ > 50% or a *p*-value for heterogeneity < 0.1, we used a random-effect model to analyze the data. Conversely, if we identified *I*^2^ < 50% or a *p*-value for heterogeneity ≥ 0.1, we used a fixed-effect model to analyze the data (15). The continuous outcomes were analyzed using a random-effect model. We transformed the median and range interquartile into mean and standard difference (SD) according to the method from Luo et al. (16) and Wan et al. (17). The dichotomous outcomes were reported as odds ratios (OR) with 95% confidence intervals (CI), while the continuous outcomes as standardized mean difference (SMD) and 95% CI. The statistical tests were two-sided, and overall effects with a *p* < 0.05 were considered to exhibit significant differences.

The subgroup analyses of the primary outcome were performed depending on types of CHD and the infusion time of dexmedetomidine (intraoperative infusion and combination of continuous intraoperative and postoperative infusion).

## Results

### Study selection

**FIGURE 1:**
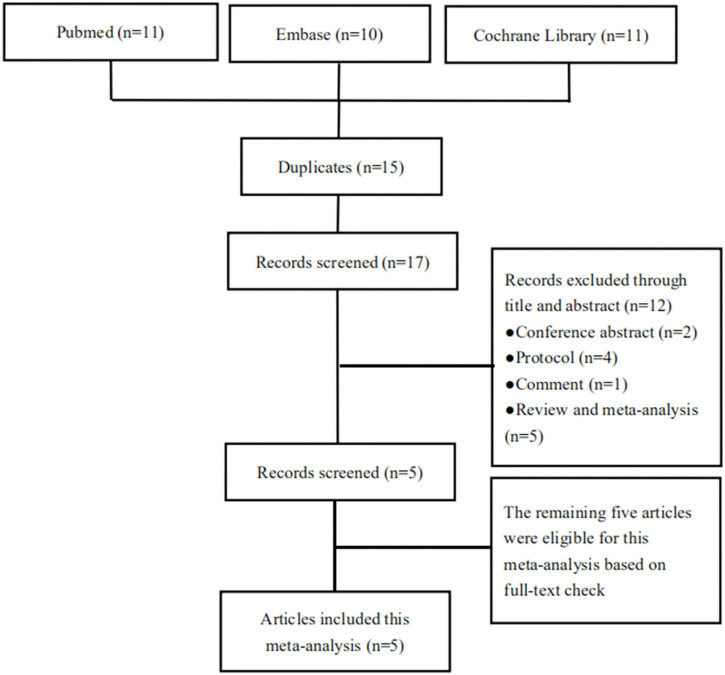
Screening process of the eligible trials.

Figure 1 presents the PRISMA flow chart for our screening process. We obtained 11 trials from Pubmed, 10 from Embase, and 11 from Cochrane Library. We removed 15 duplicate trials and excluded 12 trials at the title-and-abstract review stage based on our exclusion criteria. Finally, we enrolled five articles including a total of 630 patients (Figure 1) (18–22).

### Study characteristics

There were five trials and 630 patients. Table 1 demonstrated the basic characteristics of the included studies. Of them, four studies were RCTs (18, 19, 21, 22), and one was retrospective observational study (20). Two studies enrolled the pediatric patients undergoing cardiac surgery due to simple and complicated CHD (19, 20). The patients in the other three studies only underwent repair of ASD or VSD (18, 21, 22). Besides, three articles presented dexmedetomidine infusion intraoperatively (18, 19, 21), and one study extended until 12 after surgery (22), and the retrospective study presented postoperative dexmedetomidine infusion within 24 h after surgery (20). One study assessed the AKI by the method of AKI Network (AKIN) (18), and the others by the method of Kidney Disease Improving Global Outcomes (KDIGO). Three articles reported duration of mechanical ventilation postoperatively (19–21). Two articles presented length of ICU stay following surgery (20, 21). The number of articles provided length of hospital stay (19) and mortality (20) after surgery was only one, respectively.

**TABLE 1:** Basic characteristics of included trials.

Author	Country	Number of patients	Male,%	Age	Weight (kg)	Surgical procedures	Dexmedetomidine infusion method	CPB time (min)	Aortic clamping time (min)	Method of AKI assessment
Jo et al. (18)	Korea	29	62.1	1–6 years	11.45	Repair of ASD or VSD	**Loading dose:** 0.5 μg/kg was infused over 10 min from anesthesia induction **Maintaining dose:** 0.5 μg/kg/h to the end of CPB	106.5	68	AKIN
Kim et al. (19)	Korea	139	62.6	< 7 years	10.05	Cardiac surgery with CPB	**Loading dose:** 1 μg/kg over 10 min after the induction of anesthesia **Maintaining dose:** 1.5 μg/kg/h during surgery, and 1 μg/kg after CPB initiation	145.1	87.8	KDIGO
Kwiatkowski et al. (20)	United States	204	57	< 18 years	7	Cardiac surgery with CPB	Infusion initiated on any dose or duration within 24 h after surgery	121	58.5	KDIGO
Ming et al. (21)	China	90	57.8	1–6 years	12.7	Repair of ASD or VSD	From the onset of anesthesia until the end of the operation: 1) 0.2μg/kg/h; 2) 0.4μg/kg/h	74.6	37.7	KDIGO
Xie et al. (22)	China	168	48.8	1–6 years	_	Repair of ASD or VSD	**Loading dose:** 0.1μg/kg at 15 min before anesthesia induction **Maintaining dose:** 0.5 μg/kg/h until 12 h after operation	107.02	67.35	KDIGO

ASD, atrial septal defect; VSD, ventricular septal defect; CPB, cardiopulmonary bypass; AKI, acute kidney injury; AKIN, acute kidney injury network; KDIGO, Kidney Disease Improving Global Outcomes.

### Study quality

We used NOS to assess the risk of bias in observational studies, and the retrospective trial obtained eight stars, indicating high quality (Supplementary Table 2). We used the Cochrane Collaboration Risk of Bias Assessment tool to assess the risk of bias in RCTs. All of included RCTs were high risk of bias, as they clearly assessed random sequence generation four studies-100%), allocation concealment (zero study-0%), blinding of participants (zero study-0%), blinding of outcome assessment (zero study-0%), incomplete outcome data (four studies-100%), and selective outcome reporting (four studies-100%) (Supplementary Figure 1). Four RCTs were found to be low quality due to the possible problems of random allocation and blindness (Supplementary Figure 2).

### Post-operative acute kidney injury

**FIGURE 2:**
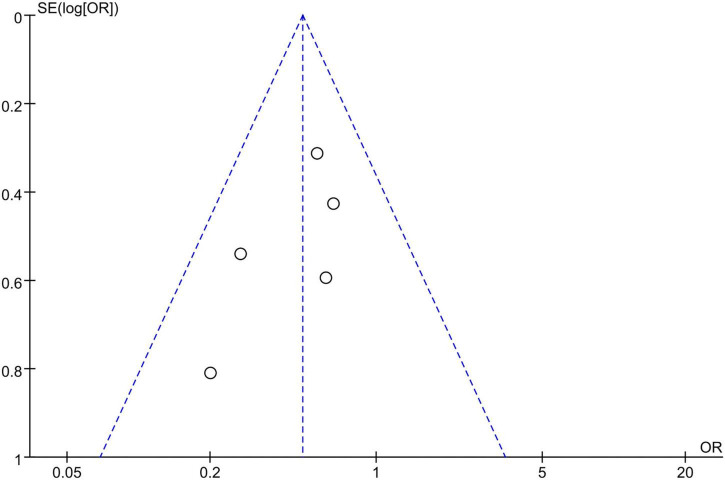
Heterogenicity of included studies by the funnel plots.

**FIGURE 3:**
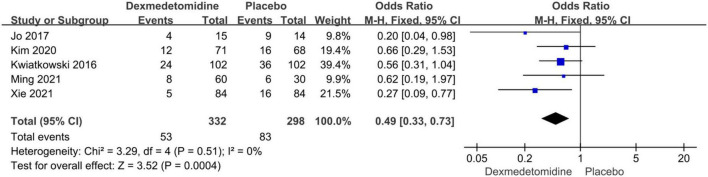
Pooled result of the incidence of AKI in pediatric patients undergoing cardiac surgery between dexmedetomidine and placebo.

The assessment (or follow-up) time and the number of patients with cardiac surgery induced AKI for each study were presented in Table 2. We used a fixed-effect model with OR to analyze the effect of dexmedetomidine infusion on the postoperative incidence of AKI in pediatric patients undergoing cardiac surgery with CPB due to low heterogeneity (*I*^2^ = 0%) (Figure 2). The pooled result demonstrated significant differences in incidence of AKI after cardiac surgery between pediatric patients with dexmedetomidine and placebo (OR = 0.49, 95% CI: [0.33, 0.73], *I*^2^ = 0%, *p* for effect = 0.0004) (Figure 3).

**TABLE 2:** Follow-up time and number of patients with AKI under different intervention methods.

Author	Assessment (or follow-up) time	Number of patients in control group	Number of patients in dexmedetomidine group	Number of patients with AKI in control group	Number of patients with AKI in dexmedetomidine group
Jo et al. (18)	Within 2 days after surgery	14	15	9	4
Kim et al. (19)	Postoperative 1-7 day	68	71	16	12
Kwiatkowski et al. (20)	After cardiac surgery	102	102	36	24
Ming et al. (21)	Within 48 h after surgery	30	0.2μg/kg/h: 30 0.4μg/kg/h: 30	6	0.2μg/kg/h: 4 0.4μg/kg/h: 4
Xie et al. (22)	Within 48 h after surgery	84	84	16	5

AKI, acute kidney injury.

**FIGURE 4:**
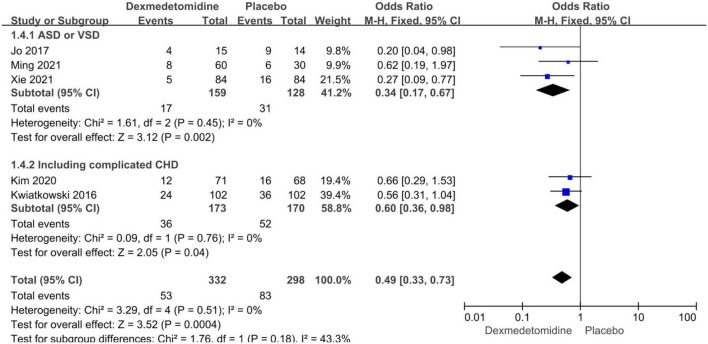
Subgroup analysis according to CHD types.

**FIGURE 5:**
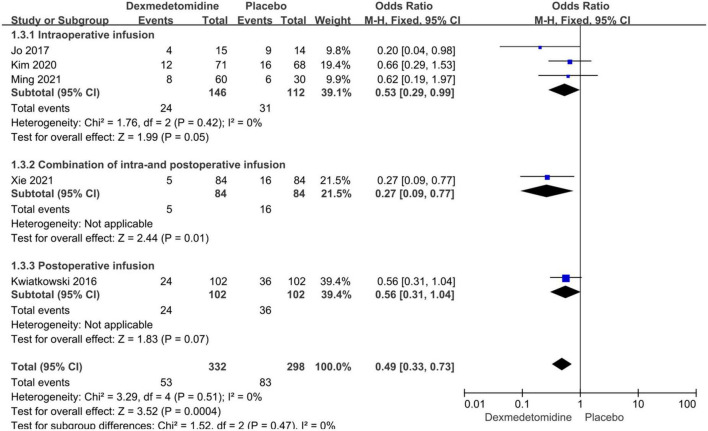
Subgroup analysis according to intervention time (intraoperative, combination of intra- and postoperative, postoperative dexmedetomidine infusion).

Subgroup analyses were performed according to the types of CHD and dexmedetomidine intervention time. Compared to placebo, dexmedetomidine significantly reduced the postoperative incidence of AKI in children undergoing correction surgery due to AVSD (OR = 0.34, 95% CI: [0.17, 0.67], *I*^2^ = 0%, *p* for effect = 0.002) or complicated CHD (OR = 0.60, 95% CI: [0.36, 0.98], *I*^2^ = 0%, *p* for effect = 0.04) (Figure 4). Additionally, compared to placebo, the pooled results did not demonstrate considerable difference in incidence of AKI after surgery in pediatric patients receiving intraoperative (OR = 0.53, 95% CI: [0.29, 0.99], *I*^2^ = 0%, *p* for effect = 0.05) or postoperative (OR = 0.56, 95% CI: [0.31, 1.04], *p* for effect = 0.07) dexmedetomidine infusion, but a significant reduction in AKI occurrence in patients with combination of intra- and postoperative dexmedetomidine infusion (OR = 0.27, 95% CI: [0.09, 0.77], *p* for effect = 0.01) (Figure 5).

### Secondary outcomes

**FIGURE 6:**
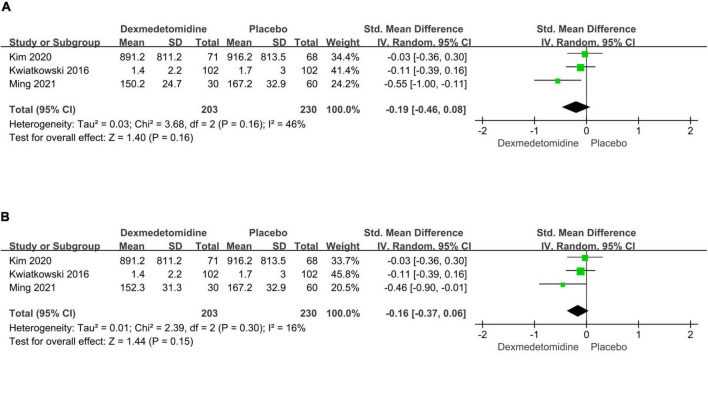
Pooled results of postoperative duration of ventilation time between dexmedetomidine and placebo groups [different dexmedetomidine dose 0.2 μg/kg/h **(A)** and 0.4 μg/kg/h **(B)** according to the study from Ming et al.].

**FIGURE 7:**
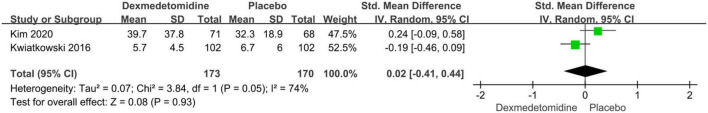
Pooled result of postoperative length of ICU stay between dexmedetomidine and placebo groups.

**FIGURE 8:**
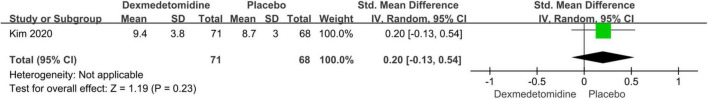
Pooled result of postoperative length of hospital stay between dexmedetomidine and placebo groups.

**FIGURE 9:**
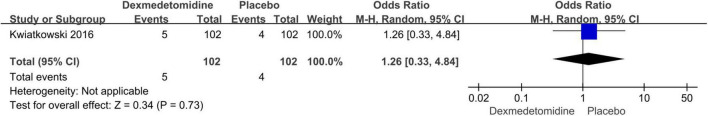
Pooled result of postoperative all-cause mortality between dexmedetomidine and placebo groups.

The important secondary outcomes of this meta-analysis were presented in Table 3. There was no significant difference in duration of mechanical ventilation (SMD: –0.19, 95% CI: –0.46 to 0.08, *p* for effect = 0.16; SMD: –0.16, 95% CI: –0.37 to 0.06, *p* for effect = 0.15) (Figure 6), length of ICU (SMD: 0.02, 95% CI: –0.41 to 0.44, *p* for effect = 0.93) (Figure 7) and hospital stay (SMD: 0.2, 95% CI: –0.13 to 0.54, *p* for effect = 0.23) (Figure 8) or in-hospital mortality (OR = 1.26, 95% CI: 0.33–4.84, *p* for effect = 0.73) (Figure 9) after surgery according to the pooled results of secondary outcomes.

**TABLE 3:** Important secondary outcomes of this meta-analysis.

Author	Duration of mechanical ventilation		Length of ICU stay		Length of hospital stay		All-cause death	
				
	Placebo	Dexmedetomidine	Placebo	Dexmedetomidine	Placebo	Dexmedetomidine	Placebo	Dexmedetomidine
Jo et al. (18)	NA	NA	NA	NA	NA	NA	NA	NA
Kim et al. (19)	780 (436–1,510) min	736 (420–1,492) min	27 (22–47) h	27 (20–70) h	8 (7–11) d	9 (7–12) d	NA	NA
Kwiatkowski et al. (20)	1 (0–4) d	1 (0–3) d	6 (3–11) d	5 (3–9) d	NA	NA	4 (4%)	5 (5%)
Ming et al. (21)	167.17 ± 32.86 min	**0.2 μ g/kg/h:** 150.20 ± 24.70 min **0.4 μ g/kg/h:** 152.30 ± 31.33 min	NA	NA	NA	NA	NA	NA
Xie et al. (22)	NA	NA	NA	NA	NA	NA	NA	NA

NA, not applicable; ICU, intensive care unit.

## Discussion

This meta-analysis included five studies and a total of 630 individuals. Of them, there were four RCTs containing 426 patients, and one retrospective study containing 204 patients. The results demonstrated that compared to placebo, dexmedetomidine infusion could significantly reduce the postoperative incidence of AKI, but the considerable difference was reflected in the pediatric patients receiving combination of intra- and postoperative dexmedetomidine infusion. Besides, there was no significant difference in duration of mechanical ventilation, length of ICU and hospital stay or in-hospital mortality after surgery between dexmedetomidine and placebo groups.

The development of all organs is not yet mature in children, especially newborns, thus they are prone to occurrence of organ dysfunction following cardiac surgery with CPB (23). Currently, there are mainly three methods to assess AKI in children—AKIN, KDIGO, and the Risk, Injury, Failure, Loss, and End-stage renal disease for pediatric use (pRIFLE) (24). In 2012, KDIGO group proposed to establish a common definition of AKI for adults and children by adjusting subtle differences between AKIN, RIFLE, and pRIFLE. The current diagnostic and staging criteria are based on abrupt varieties in serum creatinine (sCr) levels and urine output (UO) as indicators of kidney impairment (25–28). The enrolled studies in this meta-analysis included two methods of AKI assessment—AKIN and KDIGO.

Studies have explored the mechanisms and risk factors of cardiac surgery-induced AKI. Because of poor autonomous regulation ability in pediatric patients, perioperative strong systemic circulation fluctuation easily occurs, and associated with acute injury for their original immature organs, such as AKI (29). Besides, systemic inflammatory response induced by cardiac surgery and CPB is also a pivotal mechanism associated with postoperative AKI occurrence in pediatric patients. Large number of inflammatory and proinflammatory factors are released into the blood circulation, and yield kidney inflammation-induced parenchyma impairment, and eventually lead to AKI (30, 31). Recently, some AKI-related biomarkers have been identified, such as urinary liver-type fatty acid binding protein, serum or urinary neutrophil gelatinase-associated lipocalin, serum kidney injury molecule-1, serum cystatin C, and serum albumin. Additionally, the potential risk factors for AKI occurrence in pediatric patients following cardiac surgery included pulmonary hypertension, cyanotic heart disease, univentricular heart, vasopressor use, CPB use, reoperation, sepsis, younger age, lower body weight, lower preoperative sCr levels, higher preoperative estimated glomerular filtration rate, higher Risk Adjustment for Congenital Heart Surgery-1 score, longer surgery time, longer CPB time, longer aortic cross-clamp time, and higher red blood cell transfusion volume. Because there are so many risk factors for pediatric AKI after pediatric cardiac surgery, it is difficult to reduce the incidence of AKI by controlling all identified risk factors (1, 32, 33).

Dexmedetomidine, a highly selective α_2_-adrenergic receptor agonist, acts as a hypnotic and sedative agent due to reduced noradrenaline release in central nervous system by mainly activating presynaptic α_2_ receptors in the locus coeruleus, thus yielding an unconscious state similar to natural sleep (34). Because of the wide presence of α_2_ receptor in other organs, such as lung and kidney, dexmedetomidine exhibits its pharmacological role in these important organs (35, 36). Animal experiment demonstrated that dexmedetomidine could attenuate renal fibrosis through α_2_-adrenergic receptor-dependent inhibition of cellular senescence after renal ischemia/reperfusion (37). Besides, dexmedetomidine also has the features of anti-inflammation, anti-oxidation and hemodynamic stability (8, 38). Therefore, dexmedetomidine has the potential function of organ protection perioperatively. Although dexmedetomidine is an off-label drug in the pediatric patients, it currently also has been widely used as an adjuvant medication of general anesthesia or postoperative sedative in clinical practice of pediatric patients (39, 40). Furthermore, studies have proved the safety of dexmedetomidine use in patients aged less than 18 years (41, 42). In recent years, some meta-analyses have comprehensively analyzed the effect of dexmedetomidine administration on incidence of AKI in pediatric patients undergoing cardiac surgery (43, 44). Considering that this topic had an important research value, we conducted this meta-analysis by updating previous published articles.

This meta-analysis demonstrated that intra- or postoperative dexmedetomidine infusion did not exhibit significant difference in the incidence of cardiac surgery-induced AKI in pediatric patients compared to placebo, while combination of intra- and postoperative administration could considerably reduce the postoperative incidence of AKI. However, this meta-analysis only included five studies (four RCTs and one retrospective study). Of them, three studies presented intraoperative dexmedetomidine infusion, and another two studies provided postoperative and combination of intra- and postoperative dexmedetomidine administration, respectively. Furthermore, all enrolled RCTs had risk of bias due to uncertain allocation concealment and blinding of outcomes assessment, and blinding of participants and personnel. Inadequate evidence and low-quality RCTs may affect the final results, the high-quality RCT with large sample size is required to further prove the results.

In our meta-analysis, several limitations should be taken into consideration. First, there were relatively few studies in this field, and most of the studies had small sample size, meanwhile presented risk of bias, thus yielding the unreliability of the results. As the number of relevant studies increases, the pooled results may vary. Second, different dose and time of dexmedetomidine infusion among the enrolled studies might also affect the synthesized result. Third, different assessment methods of AKI may influence the pooled results. The study from Jo et al. assessed AKI by AKIN, and the others by KDIGO guidelines. Fourth, this meta-analysis only selected the published studies, the pooled results might be different if the data from gray studies were also enrolled. Fifth, dexmedetomidine infusion time was until 12 h after surgery in the study of combination of intra- and postoperative intervention. We are not aware whether a change in continuous infusion time following surgery will affect the final results.

## Conclusion

Compared to placebo, dexmedetomidine infusion could significantly decrease the incidence of AKI in pediatric patients undergoing cardiac surgery with CPB. But the statistical difference was only presented in the study with combination of intra- and postoperative dexmedetomidine infusion. Besides, there was no significant difference in duration of mechanical ventilation, length of ICU and hospital stay or in-hospital mortality after surgery between dexmedetomidine and placebo groups based on the analyses of the secondary outcomes. However, considering that there are only five articles with small sample size and most of them have higher risk of bias, the pooled result may be unreliable. Because this topic has important clinical implications, the further updated meta-analysis is required to follow the results with the increasing number of relevant studies.

## Data availability statement

The original contributions presented in this study are included in the article/Supplementary material, further inquiries can be directed to the corresponding author/s.

## Author contributions

HW and CZ independently assessed the quality of included studies and performed the screening process for titles and abstracts. YL and YJ performed the screening process for full texts. SY and YL were responsible for extracting the data. HW, JW, and FY were responsible for adjusting data discrepancies. JW and FY independently assessed the quality of included studies and supervised the whole process and ensured the effectiveness of the meta-analysis. YJ conducted the statistical analysis and made the figures and tables. HW prepared the manuscript. All authors read and approved the submission of the final manuscript.

## Supplementary material

Supplementary Figure 1 Risk of bias summary: review authors’ judgments about each risk of bias item for each included study.

Supplementary Figure 2 Risk of bias graph: review authors’ judgments about each risk of bias item presented as percentages across all included studies.
