# Supplementary material for: Dexmedetomidine and acute kidney injury following cardiac surgery in pediatric patients—An updated systematic review and meta-analysis
Source: Front Cardiovasc Med. 2022 Aug 24;9:938790. doi: 10.3389/fcvm.2022.938790 (PMC9448974; doi:10.3389/fcvm.2022.938790)
Supplement: Supplementary file 1 [file Table_1.DOC]

| **Section/topic** | **#** | **Checklist item** | **Reported on page #** |
| --- | --- | --- | --- |
| **TITLE** | | |  |
| Title | 1 | Perioperative dexmedetomidine infusion and acute kidney injury following cardiac surgery in pediatric patients-An updated systematic review and meta-analysis | P 1 |
| **ABSTRACT** | | |  |
| Structured summary | 2 | **Abstract**  **Background:** Acute kidney injury (AKI) is a common postoperative complication in pediatric patients undergoing cardiac surgery and associated with poor outcomes. Dexmedetomidine has the pharmacological features of organ protection in cardiac surgery patients. The aim of this meta-analysis is to investigate the effect of perioperative dexmedetomidine infusion on AKI incidence after cardiac surgery in pediatric patients.  **Methods:** The databases of Pubmed, Embase, and Cochrane Library were searched until April 24, 2022 following the Preferred Reporting Items for Systematic Reviews  and Meta-Analyses (PRISMA) guidelines. RevMan 5.3 was used to perform statistical analyses.  **Results:** Five relevant trials with a total of 630 patients were included. The pooled result using fixed-effects model with OR demonstrated significant difference in AKI incidence between patients with dexmedetomidine and placebo (OR=0.49, 95% CI: [0.33, 0.73], I^2^=0%, *p* for effect=0.0004). The subgroup analyses were performed based on CHD types and dexmedetomidine intervention time. The pooled results did not demonstrate considerable difference in AKI incidence in pediatric patients receiving intraoperative (OR=0.53, 95% CI: [0.29, 0.99], I^2^=0%, *p* for effect=0.05) or postoperative dexmedetomidine infusion (OR=0.56, 95% CI: [0.31, 1.04], *p* for effect=0.07), but a significant difference in patients receiving combination of intra- and postoperative dexmedetomidine infusion (OR=0.27, 95% CI: [0.09, 0.77], *p* for effect=0.01). Besides, there was no significant difference in postoperative mechanical ventilation time (SMD: -0.19, 95% CI: -0.46 to 0.08, *p* for effect=0.16; SMD: -0.16, 95% CI: -0.37 to 0.06, *p* for effect=0.15), ICU stay time (SMD: 0.02, 95% CI: -0.41 to 0.44, *p* for effect=0.93), hospital stay time (SMD: 0.2, 95% CI: -0.13 to 0.54, *p* for effect=0.23) and mortality (OR=1.26, 95% CI: 0.33 to 4.84, *p* for effect=0.73) according to the pooled results of the secondary outcomes.  **Conclusion:** Compared to placebo, perioperative dexmedetomidine infusion could significantly reduce the postoperative AKI incidence in pediatric patients undergoing cardiac surgery with CPB, but the considerable difference was reflected in the pediatric patients receiving combination of intra- and postoperative dexmedetomidine infusion. Besides, there was no significant difference in postoperative mechanical ventilation time, ICU stay time, hospital stay time and mortality. | P 1-2 |
| **INTRODUCTION** | | |  |
| Rationale | 3 | Acute kidney injury (AKI) is a common postoperative complication in pediatric patients undergoing cardiac surgery with cardiopulmonary bypass (CPB) due to congenital heart disease (CHD). AKI incidence ranges from 20% to 86% depending on different diagnostic tools and medical centers, and can be even higher among neonates. Dexmedetomidine is a highly selective α_2_ -adrenergic receptor agonist, and it also has the pharmacological features of sedation, analgesia, systemic circulation stability and anti-inflammatory response [8]. Therefore, dexmedetomidine has the potential function of nephroprotection in pediatric patients undergoing cardiac surgery. | P 2 |
| Objectives | 4 | The primary aim of this meta-analysis was to investigate the effect of perioperative dexmedetomidine infusion on AKI incidence in pediatric patients undergoing cardiac surgery with CPB. Additionally, we also evaluated the association between perioperative dexmedetomidine infusion and the postoperative mechanical ventilation time, ICU stay, hospital stay, and all-cause mortality as the secondary outcomes. | P 4 |
| **METHODS** | | |  |
| Protocol and registration | 5 | No registration |  |
| Eligibility criteria | 6 | The inclusion criteria were 1) participants aged younger than 18 years; 2) patients undergoing cardiac surgery; and 3) articles reporting the effect of dexmedetomidine on AKI. The exclusion criteria were: 1) duplicate articles; 2) participants older than 18 years old; 3) review or meta-analysis; 4) articles published as an abstract, letter, case report, basic research, editorial, note, method, or protocol; 5) articles presented in a non-English language; 6) studies without a specific number of patients with dexmedetomidine (observational studies) and/or AKI. | P 3 |
| Information sources | 7 | We searched the databases of Pubmed, Embase, and Cochrane Library using the PICOS (Population, Intervention, Comparison, Outcome, Study design) method. Our last search was completed on April 24, 2022. | P 2 |
| Search | 8 | The search terms included “pediatric” OR “pediatrics” OR “child” OR “children” OR “infant” OR “infants” OR neonate” OR “neonates” OR “newborn” OR “newborns” OR “teenager” OR “teenagers” AND “cardiac surgery” OR “cardiac operation” OR “cardiac surgeries” OR “heart surgery” OR “heart surgeries” OR “heart operation” AND “acute kidney injury” OR “acute renal injury” OR “acute kidney failure” OR “acute renal failure” OR “acute kidney insufficiency” OR “acute renal insufficiency” AND “dexmedetomidine” OR “MPV-1440” OR “MPV 1440” OR “MPV1440” OR “Precedex” OR “dexmedetomidine Hydrochloride” and the search scope was “title and abstract.” Because we sought to examine all studies about the effect of dexmedetomidine on AKI incidence in pediatric patients undergoing cardiac surgery, we did not constrain the search terms for study designs. | P2- 3 |
| Study selection | 9 | The inclusion criteria were 1) participants aged younger than 18 years; 2) patients undergoing cardiac surgery; and 3) articles reporting the effect of dexmedetomidine on AKI. The exclusion criteria were: 1) duplicate articles; 2) participants older than 18 years old; 3) review or meta-analysis; 4) articles published as an abstract, letter, case report, basic research, editorial, note, method, or protocol; 5) articles presented in a non-English language; 6) studies without a specific number of patients with dexmedetomidine (observational studies) and/or AKI. | P 3 |
| Data collection process | 10 | Two authors were independently responsible for reviewing the titles, abstracts or both and summarized the data of the included literatures. Another three authors were in charge of the data discrepancy adjustment. | P 3 |
| Data items | 11 | Two authors were responsible for extracting the following information: 1) authors; 2) publication year; 3) country of publication; 4) total number of participants in each study; 4) percentage of males; 5) age range of all the participants; 6) weight; 7) procedures that the participants underwent; 8) methods of dexmedetomidine administration; 9) duration of CPB; 10) duration of aortic clamping; 11) assessment time of AKI; 12) number of patients with or without dexmedetomidine; 13) assessment (or follow-up) time; 14) number of patients with and without AKI; 15) postoperative ventilation time; 16) postoperative ICU stay time; 17) postoperative hospital stay time; 18) number of dead patients during the follow-up time. Another two authors were responsible for adjusting data discrepancies. | P 4 |
| Risk of bias in individual studies | 12 | Two authors independently assessed the quality of included studies. The Cochrane Collaboration Risk of Bias Assessment tool was used to assess the risk of bias of all included RCTs, and the Newcastle-Otawa Quality Assessment Scale (NOS) was used to assess the bias risk of observational trials. If the two authors had the different assessment results, they consulted the third or the forth one. Eventually, we reached consensus. | P 3 |
| Summary measures | 13 | The dichotomous outcome were reported as odds ratios (OR) with 95% confidence interval (CI). The statistical tests were two-sided and *p* value for overall effect <0.05 was considered significant differences. | P 4 |
| Synthesis of results | 14 | Review Manager (RevMan) version 5.3 (Cochrane collaboration, Oxford, UK) was used to perform statistical analyses. We assessed the heterogeneity of included studies using the values of I^2^ and the Mantel-Haenszel chi-square test (*p*-value for heterogeneity). The values of I^2^<40%, I^2^=40–60%, and I^2^>60% indicated low, moderate, and high heterogeneity, respectively (14). If we identified I^2^>50% or a *p*-value for heterogeneity<0.1, we used a random-effect model to analyze the data. Conversely, if we identified I^2^ <50% or a *p*-value for heterogeneity≥0.1, we used a fixed-effect model to analyze the data (15). The continuous outcomes were analysed using a random-effect model. We transformed the median and range interquartile into mean and standard difference (SD) according to the methods from Luo et al. and Wan et al. (16,17). The dichotomous outcomes were reported as odds ratios (OR) with 95% confidence intervals (CI), while the continuous outcomes as standardized mean difference (SMD) and 95% CI. The statistical tests were two-sided, and overall effects with a *p*<0.05 were considered to exhibit significant differences.  The subgroup analyses of the primary outcome were performed depending on types of CHD and the infusion time of dexmedetomidine (intraoperative infusion and combination of continuous intraoperative and postoperative infusion). | P 4 |

Page 1 of 2

| **Section/topic** | **#** | **Checklist item** | **Reported on page #** |
| --- | --- | --- | --- |
| Risk of bias across studies | 15 | We used NOS to assess the risk of bias in observational studies, and the retrospective trial obtained seven stars, indicating high quality. We used the Cochrane Collaboration Risk of Bias Assessment tool to assess the risk of bias in RCTs. All of included RCTs were high risk of bias, as they clearly assessed random sequence generation four studies-100%), allocation concealment (zero study-0%), blinding of participants (zero study-0%), blinding of outcome assessment (zero study-0%), incomplete outcome data (four studies-100%), and selective outcome reporting (four studies-100%) (Supplementary Figure 1). Four RCTs were found to be low quality due to the possible problems of random allocation and blindness. | P5 |
| Additional analyses | 16 | The subgroup analyses was performed depending on types of CHD and the infusion time of dexmedetomidine (intraoperative infusion and combination of continuous intraoperative and postoperative infusion). | P4 |
| **RESULTS** | | |  |
| Study selection | 17 | See Figure 1 | P 4 |
| Study characteristics | 18 | For each study, present characteristics for which data were extracted (e.g., study size, PICOS, follow-up period) and provide the citations. | P 4-5 |
| Risk of bias within studies | 19 | Present data on risk of bias of each study and, if available, any outcome level assessment (see item 12). | P 3 |
| Results of individual studies | 20 | For all outcomes considered (benefits or harms), present, for each study: (a) simple summary data for each intervention group (b) effect estimates and confidence intervals, ideally with a forest plot. | P 6 |
| Synthesis of results | 21 | The assessment (or follow-up) time and the number of patients with cardiac surgery induced AKI for each study were presented in Table 2. We used a fixed-effect model with OR to analyse the effect of dexmedetomidine infusion on postoperative AKI incidence in pediatric patients undergoing cardiac surgery with CPB due to low heterogeneity (I^2^=0%) (Figure 2). The pooled result demonstrated significant differences in incidence of AKI after cardiac surgery between pediatric patients with dexmedetomidine and placebo (OR=0.49, 95% CI: [0.33, 0.73], I^2^=0%, *p* for effect=0.0004. | P 5 |
| Risk of bias across studies | 22 | Present results of any assessment of risk of bias across studies (see Item 15). | P 5 |
| Additional analysis | 23 | The subgroup analyses were performed according to the types of CHD and dexmedetomidine intervention time. Compared to placebo, dexmedetomidine significantly reduced the postoperative AKI incidence in children undergoing correction surgery due to AVSD (OR=0.34, 95% CI: [0.17, 0.67], I^2^=0%, *p* for effect=0.002) or complicated CHD (OR=0.60, 95% CI: [0.36, 0.98], I^2^=0%, *p* for effect=0.04) (Figure 4). Additionally, compared to placebo, the pooled results did not demonstrate considerable difference in incidence of AKI after surgery in pediatric patients receiving intraoperative (OR=0.53, 95% CI: [0.29, 0.99], I^2^=0%, *p* for effect=0.05) or postoperative (OR=0.56, 95% CI: [0.31, 1.04], *p* for effect=0.07) dexmedetomidine infusion, but a significant reduction in AKI occurrence in patients with combination of intra- and postoperative dexmedetomidine infusion (OR=0.27, 95% CI: [0.09, 0.77], *p* for effect=0.01) (Figure 5).  **The secondary outcomes**  There were no significant difference in mechanical ventilation time (SMD: -0.19, 95% CI: -0.46 to 0.08, *p* for effect=0.16; SMD: -0.16, 95% CI: -0.37 to 0.06, *p* for effect=0.15) (Figure 6), ICU stay time (SMD: 0.02, 95% CI: -0.41 to 0.44, *p* for effect=0.93) (Figure 7), hospital stay time (SMD: 0.2, 95% CI: -0.13 to 0.54, *p* for effect=0.23) (Figure 8) and mortality (OR=1.26, 95% CI: 0.33 to 4.84, *p* for effect=0.73) (Figure 9) according to the pooled results of secondary outcomes. | P 5-6 |
| **DISCUSSION** | | |  |
| Summary of evidence | 24 | This meta-analysis included five studies and a total of 630 individuals. Of them, there were four RCTs containing 426 patients, and one retrospective study containing 204 patients. The results demonstrated that compared to placebo, perioperative dexmedetomidine infusion could significantly reduce the postoperative AKI incidence, but the considerable difference was reflected in the pediatric patients receiving combination of intra- and postoperative dexmedetomidine infusion. | P 5 |
| Limitations | 25 | In our meta-analysis, several limitations should be taken into consideration. First, there were relatively few studies in this field, and most of the studies had small sample size, meanwhile presented risk of bias, thus yielding the unreliability of the result. As the number of relevant studies increases, the pooled results may vary. Second, different dose and time of dexmedetomidine infusion among the enrolled studies might also affect the synthesized result. Third, different assessment methods of AKI may influence the pooled results. The study from Jo assessed AKI by AKIN, and the others by KDIGO guidelines. Fourth, this meta-analysis only selected the published studies, the pooled results might be different if the data from gray studies were enrolled. Fifth, dexmedetomidine infusion time was until 12 h after surgery in the study of combination of intra- and postoperative intervention. We are not aware whether a change in continuous infusion time following surgery will affect the final results. | P 7 |
| Conclusions | 26 | Compared to placebo, perioperative dexmedetomidine infusion could significantly decrease the incidence of AKI in pediatric patients undergoing cardiac surgery with CPB. But the statistical difference was only presented in the study with combination of intra- and postoperative dexmedetomidine infusion. Besides, we did not obtain the significant difference in postoperative mechanical ventilation time, ICU stay time, hospital stay time and all-cause mortality between dexmedetomidine and placebo groups based on the analyses of the secondary outcomes. However, considering that there are only five articles with small sample size and most of them have higher risk of bias, the pooled result may be unreliable. Because this topic has important clinical implications, the further updated meta-analysis is required to follow the results with the increasing number of relevant studies. | P 7 |
| **FUNDING** | | |  |
| Funding | 27 | No funder. |  |

*From:*  Moher D, Liberati A, Tetzlaff J, Altman DG, The PRISMA Group (2009). Preferred Reporting Items for Systematic Reviews and Meta-Analyses: The PRISMA Statement. PLoS Med 6(7): e1000097. doi:10.1371/journal.pmed1000097

For more information, visit: **www.prisma-statement.org**.

Page 2 of 2
