# Supplementary material for: Dexmedetomidine and acute kidney injury following cardiac surgery in pediatric patients—An updated systematic review and meta-analysis
Source: Front Cardiovasc Med. 2022 Aug 24;9:938790. doi: 10.3389/fcvm.2022.938790 (PMC9448974; doi:10.3389/fcvm.2022.938790)
Supplement: Supplementary file 2 [file Table_2.DOC]

Supplementary Table 2. Bias risk of observational studies (retrospective and prospective) by the Newcastle-Ottawa Quality Assessment Scale

| **Study** | **Selection** | | | | **Comparability** | **Outcome** | | | **Total score** |
| --- | --- | --- | --- | --- | --- | --- | --- | --- | --- |
|  | **Representativenes of exposed cohort** | **Selection of unexposed**  **cohort** | **Ascertainment of exposure** | **Outcome of interest** |  | **Assessment of outcome** | **Follow-up long enough for outcomes to occur** | **Adequacy**  **of follow-up** |  |
| Kwiatkowski 2021 | * | * | * | * | * | * | * | * | 8 |
