# Supplementary figures and images for: Dexmedetomidine and acute kidney injury following cardiac surgery in pediatric patients—An updated systematic review and meta-analysis
Source: Front Cardiovasc Med. 2022 Aug 24;9:938790. doi: 10.3389/fcvm.2022.938790 (PMC9448974; doi:10.3389/fcvm.2022.938790)

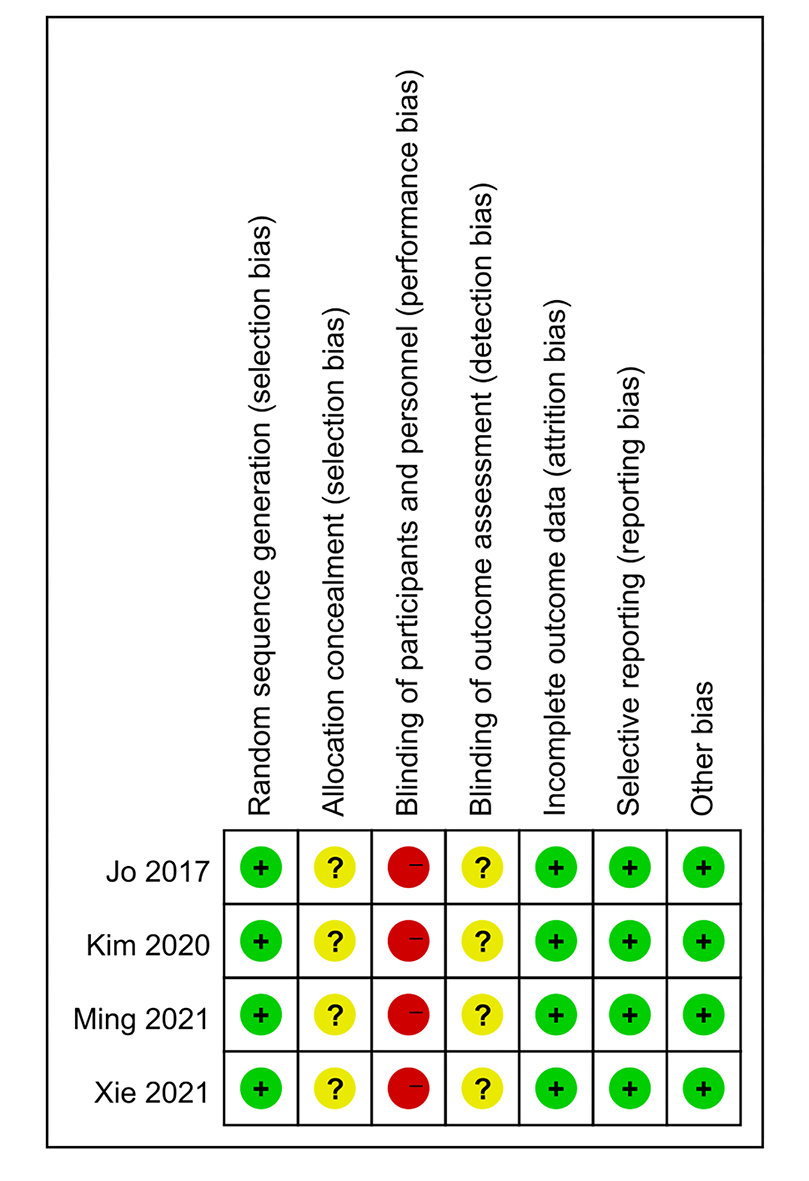

Supplement: Supplementary Figure 1 — Risk of bias summary: review authors’ judgments about each risk of bias item for each included study. [file Image_1.TIF]

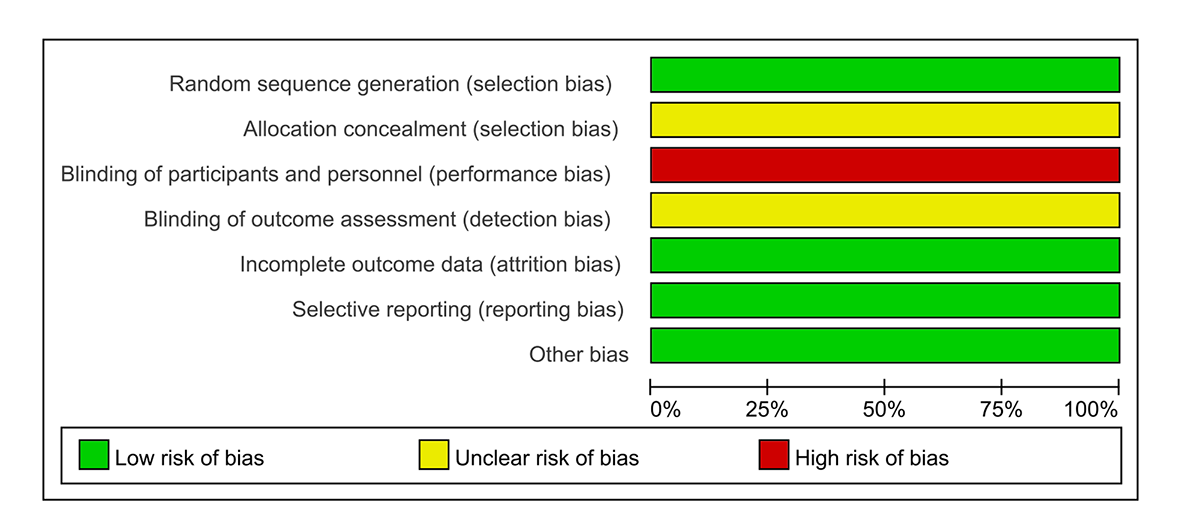

Supplement: Supplementary Figure 2 — Risk of bias graph: review authors’ judgments about each risk of bias item presented as percentages across all included studies. [file Image_2.TIF]
